# Author Correction: Single-cell brain organoid screening identifies developmental defects in autism

**DOI:** 10.1038/s41586-023-06836-5

**Published:** 2023-11-14

**Authors:** Chong Li, Jonas Simon Fleck, Catarina Martins-Costa, Thomas R. Burkard, Jan Themann, Marlene Stuempflen, Angela Maria Peer, Ábel Vertesy, Jamie B. Littleboy, Christopher Esk, Ulrich Elling, Gregor Kasprian, Nina S. Corsini, Barbara Treutlein, Juergen A. Knoblich

**Affiliations:** 1 Institute of Molecular Biotechnology of the Austrian Academy of Science (IMBA), Vienna, Austria; 2 Department of Biosystems Science and Engineering, ETH Zürich, Basel, Switzerland; 3 Department of Radiodiagnostics, Medical University of Vienna, Vienna, Austria; 4 Institute of Molecular Biology, University of Innsbruck, Innsbruck, Austria; 5 Department of Neurology, Medical University of Vienna, Vienna, Austria

**Keywords:** Stem cells, Stem-cell biotechnology, Development of the nervous system

Correction to: *Nature* 10.1038/s41586-023-06473-y Published online 13 September 2023

In the version of the article initially published, there was an error in Extended Data Fig. 4c–e where the colour scheme and gene names were mislabelled. This has now been corrected in the HTML and PDF versions of the article. The original and revised figures are shown in the Supplementary Information accompanying this amendment.

### Supplementary information

Original and revised Extended Data Fig. 4

