# Supplementary material for: Single-cell brain organoid screening identifies developmental defects in autism
Source: Nature. 2023 Sep 13;621(7978):46. doi: 10.1038/s41586-023-06473-y (PMC10499611; doi:10.1038/s41586-023-06473-y)
Supplement: Supplementary file 2 — Reporting Summary [file 41586_2023_6473_MOESM2_ESM.pdf]

nature portfolio

Corresponding author(s): Chong Li, Barbara Treutlein, Juergen A. Knoblich

Last updated by author(s): Jun 26, 2023

Reporting Summary

Nature Portfolio wishes to improve the reproducibility of the work that we publish. This form provides structure for consistency and transparency in reporting. For further information on Nature Portfolio policies, see our Editorial Policies and the Editorial Policy Checklist.

Statistics

For all statistical analyses, confirm that the following items are present in the figure legend, table legend, main text, or Methods section.

n/a	Confirmed	
☐	☒	The exact sample size (*n*) for each experimental group/condition, given as a discrete number and unit of measurement
☐	☒	A statement on whether measurements were taken from distinct samples or whether the same sample was measured repeatedly
☐	☒	The statistical test(s) used AND whether they are one- or two-sided<br>*Only common tests should be described solely by name; describe more complex techniques in the Methods section.*
☐	☒	A description of all covariates tested
☐	☒	A description of any assumptions or corrections, such as tests of normality and adjustment for multiple comparisons
☐	☒	A full description of the statistical parameters including central tendency (e.g. means) or other basic estimates (e.g. regression coefficient) AND variation (e.g. standard deviation) or associated estimates of uncertainty (e.g. confidence intervals)
☐	☒	For null hypothesis testing, the test statistic (e.g. F, t, r) with confidence intervals, effect sizes, degrees of freedom and P value noted<br>*Give P values as exact values whenever suitable.*
☒	☐	For Bayesian analysis, information on the choice of priors and Markov chain Monte Carlo settings
☒	☐	For hierarchical and complex designs, identification of the appropriate level for tests and full reporting of outcomes
☐	☒	Estimates of effect sizes (e.g. Cohen's *d*, Pearson's *r*), indicating how they were calculated

Our web collection on statistics for biologists contains articles on many of the points above.

Software and code

Policy information about availability of computer code

Data collection	CellSense software was used for microscopic images acquisition.<br>FACSDiva software was used for FACS data acquisition.
Data analysis	FlowJO was used for FACS data analysis.<br>FIJI was used for microscopic image processing.

For manuscripts utilizing custom algorithms or software that are central to the research but not yet described in published literature, software must be made available to editors and reviewers. We strongly encourage code deposition in a community repository (e.g. GitHub). See the Nature Portfolio guidelines for submitting code & software for further information.

Data

Policy information about availability of data

All manuscripts must include a data availability statement. This statement should provide the following information, where applicable:

- Accession codes, unique identifiers, or web links for publicly available datasets
- A description of any restrictions on data availability
- For clinical datasets or third party data, please ensure that the statement adheres to our policy

Raw sequencing data will be deposited on ArrayExpress. Processed Seurat objects were deposited on Zenodo (DOI: 10.5281/zenodo.7083558). The Pando R package is available on GitHub (https://github.com/quadbiolab/Pando). Other custom code used in the analyses is deposited on GitHub (https://github.com/quadbiolab/ASD_CHOOSE).

Field-specific reporting

Please select the one below that is the best fit for your research. If you are not sure, read the appropriate sections before making your selection.

☒ Life sciences ☐ Behavioural & social sciences ☐ Ecological, evolutionary & environmental sciences

For a reference copy of the document with all sections, see nature.com/documents/nr-reporting-summary-flat.pdf

Life sciences study design

All studies must disclose on these points even when the disclosure is negative.

Sample size	For scRNAseq screening of the 4-month-old CHOOSE organoids, we analyzed 14 10X genomics 3' GEX libraries. Each library consists of a pool of 3-7 organoids, and in total 65 organoids were sampled.<br>For bulk analysis of gRNA representation and clone information, 24 samples (50-150K cells input) were used.<br>For individual validation of over-proliferation and depletion, 3 or 5 pools of organoids (3 organoids each pool) were analyzed.<br>For individual validation of intermediate progenitors and interneuron precursor cells, at least 4 organoids were analyzed for each gene.<br>For phenotypic characterization of ARID1B patient iPSCs-derived organoids, a minimum of 3 independent batches and 13 organoids for each cell line were subjected to analyses. No statistical methods were used to predetermine sample sizes.
Data exclusions	No data were excluded.
Replication	For the CHOOSE screen, 65 cerebral organoids were sampled from 3 batches. 14 scRNA-seq libraries (biological replicates) were prepared and analyzed. 3-7 organoids were included within each library. For each ARID1B iPSC line, a minimum of 13 organoids from 3 batches were repeated. For individual validation of over-proliferation and depletion, intermediate progenitors and interneuron precursors, replicates were done from 2 batches of organoids. The individual "n" values were indicated either by displaying all data points or in the figure legend.
Randomization	Samples were not randomized to each experimental groups.
Blinding	Investigators were not blinded in this study.

Reporting for specific materials, systems and methods

We require information from authors about some types of materials, experimental systems and methods used in many studies. Here, indicate whether each material, system or method listed is relevant to your study. If you are not sure if a list item applies to your research, read the appropriate section before selecting a response.

Materials & experimental systems

n/a	Involved in the study	
☐	☒	Antibodies
☐	☒	Eukaryotic cell lines
☒	☐	Palaeontology and archaeology
☒	☐	Animals and other organisms
☐	☒	Human research participants
☒	☐	Clinical data
☒	☐	Dual use research of concern

Methods

n/a	Involved in the study	
☒	☐	ChIP-seq
☐	☒	Flow cytometry
☐	☒	MRI-based neuroimaging

Antibodies

Antibodies used	DLX2 (Santa Cruz, cat. no. SC393879, 1:100), OLIG2 (Abcam, cat. no. ab109186, 1:100), SOX2 (R&D, cat. no. MAB2018, 1:500), FOXG1 (Abcam, cat. no. ab18259, 1:200), EOMES (R&D, cat. no. AF6166, 1: 200), ARID1B (cell signaling, cat. no. 92964, 1:100), ADNP (ThermoFisher, cat. no. 702911, 1:250), BCL11A (abcam, cat. no. 191401, 1:250), PHF3 (Sigma, cat. no. HPA024678, 1:250), SMARCC2 (ThermoFisher, cat. no. PA5-54351, 1:250), KMT2C (Sigma, cat. no. HPA074736, 1:250), Alexa 488, 568, 647 conjugated secondary antibodies raised in donkey (ThermoFisher, 1:250)
Validation	DLX2 (Santa Cruz, cat. no. SC393879, mouse, 1:100) has been validated by the company and used in 3 scientific literatures.<br>OLIG2 (Abcam, cat. no. ab109186, rabbit, 1:100) has been validated by the company and used in 94 scientific literatures.<br>SOX2 (R&D, cat. no. MAB2018, 1:500) has been validated by the company and used in 145 scientific literatures.<br>FOXG1 (Abcam, cat. no. ab18259, rabbit, 1:200) has been validated by the company and used in 88 scientific literatures.<br>ARID1B (cell signaling, cat. no. 92964, mouse, 1:100), has been validated by the company and used in 9 scientific literatures.<br>ADNP (aThermoFisher, cat. no. 702911, 1:250) has been validated by the company.<br>BCL11A (abcam, cat. no. 191401, rabbit, 1:250), has been validated by the company and used in 2 scientific literatures.<br>PHF3 (Sigma, cat. no. HPA024676, rabbit, 1:250), developed and validated by the Human Protein Atlas (HPA) project.<br>SMARCC2 (ThermoFisher, cat. no. PA5-54351, rabbit, 1:250), has been validated by the company.

KMT2C (Sigma, cat. no. HPA074736, rabbit, 1:250), developed and validated by the Human Protein Atlas (HPA) project.

Eukaryotic cell lines

Policy information about cell lines

Cell line source(s)	Patient induced pluripotent stem cells were reprogrammed in IMBA SCCF from blood (PBMCs).<br>Inducible eCas9 cell line was previously generated in the lab using H9 cell line (obtained from WiCell).<br>HEK293T and NIH3T3 cell lines were purchased from ATCC.<br>Plat-E cell line was purchased from CellBiolabs.
Authentication	All stem cell lines used were authenticated using a short tandem repeat (STR) assay, tested for genomic integrity using SNP array genotyping.<br>HEK293T, NIH3T3 and Plat-E cell lines were not authenticated.
Mycoplasma contamination	All stem cell lines were tested negative for mycoplasma.<br>HEK293T, NIH3T3 and Plat-E cell lines were not tested for mycoplasma.
Commonly misidentified lines<br>(See ICLAC register)	No ICLAC lines were used.

Human research participants

Policy information about studies involving human research participants

Population characteristics	Women with singleton pregnancies undergoing fetal MRI at a tertiary care center from January 2016 and December 2021 were retrospectively reviewed.
Recruitment	A retrospective review of patient records was performed and a patient with a positive genetic testing report for ARID1B mutation was selected.
Ethics oversight	The study was approved by the institutional ethics board (Medical University of Vienna).

Note that full information on the approval of the study protocol must also be provided in the manuscript.

Flow Cytometry

Plots

Confirm that:

☒ The axis labels state the marker and fluorochrome used (e.g. CD4-FITC).

☒ The axis scales are clearly visible. Include numbers along axes only for bottom left plot of group (a 'group' is an analysis of identical markers).

☒ All plots are contour plots with outliers or pseudocolor plots.

☒ A numerical value for number of cells or percentage (with statistics) is provided.

Methodology

Sample preparation	Dissociation of organoids samples are described in the methods.
Instrument	BD FACSAria™ III
Software	FACSDivam FlowJo
Cell population abundance	Cell population abundance was indicated in Extended Data Fig. 2a. 2.5% of cells are positive for GFP.
Gating strategy	FSC/SSC were used for identify cell population. GFP, Tomato and Alexa 700 were used to select cell populations for scRNA seq.

☒ Tick this box to confirm that a figure exemplifying the gating strategy is provided in the Supplementary Information.

Magnetic resonance imaging

Experimental design

Design type	Cross-sectional fetal MRI

Design specifications	Patients undergoing fetal MRI were subjected to two fetal MRI scans during the second and third trimester. MRI was performed without sedation and without contrast media.
Behavioral performance measures	No behavioral parameters were evaluated. MRI data was used to calculate regional sub-volumes within the fetal brain throughout gestation.

Acquisition

Imaging type(s)	Structural
Field strength	1.5 and 3.0 T
Sequence & imaging parameters	Fetal MRI was conducted in accordance with the ISUOG guidelines of 2017. For fetal brain volumetry, T2-weigthed TSE sequences were used with a slice thickness of 3-4 mm, an echo time of 140 ms, and a field of view of 230 mm.
Area of acquisition	The fetus was investigated from head to toe, however in post-processing, only scans of the fetal head were analysed and quantified

Diffusion MRI ☐ Used ☒ Not used

Preprocessing

Preprocessing software	Horos, ITK-SNAP
Normalization	Segmentation of cerebral regions of interest was performed by nonrigid mapping of a publicly-available, spatiotemporal, anatomical fetal brain atlas for each individual case (1). To account for inaccuracies in ultrasound-based estimation of the exact date of conception as well as individual variability in neuronal development, we also included atlases that covered the prior and consecutive weeks of estimated gestational age for each case and merged them using a label fusion technique(2).<br><br>1. Gholipour A, Rollins CK, Velasco-Annis C, Ouaalam A, Akhondi-Asl A, Afacan O, et al. A normative spatiotemporal MRI atlas of the fetal brain for automatic segmentation and analysis of early brain growth. Sci Rep. 2017 Mar 28;7(1):476.<br>2. Wang H, Suh JW, Das SR, Pluta JB, Craige C, Yushkevich PA. Multi-Atlas Segmentation with Joint Label Fusion. IEEE Trans Pattern Anal Mach Intell. 2013 Mar;35(3):611–23.
Normalization template	See above.
Noise and artifact removal	The investigated woman was instructed with respiratory commands during the MRI scan. Additionally, during post-processing, data was denoised using the methodology described by Coupe P et al.<br><br>Coupe P, Yger P, Prima S, Hellier P, Kervrann C, Barillot C. An optimized blockwise nonlocal means denoising filter for 3-D magnetic resonance images. IEEE Trans Med Imaging. 2008 Apr;27(4):425–41.
Volume censoring	Automated brain masking was conducted as previously described by Ebner M et al.<br><br>Ebner, M. et al. An automated framework for localization, segmentation and super-resolution reconstruction of fetal brain MRI. Neuroimage 206, 116324 (2020).

Statistical modeling & inference

Model type and settings	Cross-sectional brain volumes of one patient were extracted and analyzed. No additional statistical analysis was performed.
Effect(s) tested	NA.

Specify type of analysis: ☐ Whole brain ☒ ROI-based ☐ Both

Anatomical location(s)	*Describe how anatomical locations were determined (e.g. specify whether automated labeling algorithms or probabilistic atlases were used).*
Statistic type for inference (See Eklund et al. 2016)	Voxel-wise analysis and calculation of respective volumes in mm^3.
Correction	NA.

Models & analysis

n/a	Involved in the study
☒	☐ Functional and/or effective connectivity
☒	☐ Graph analysis
☒	☐ Multivariate modeling or predictive analysis
