# Supplementary material for: Author Correction: Single-cell brain organoid screening identifies developmental defects in autism
Source: Nature. 2023 Nov 14;623(7989):E20. doi: 10.1038/s41586-023-06836-5 (PMC10686816; doi:10.1038/s41586-023-06836-5)
Supplement: Supplementary file 1 — Original and revised Extended Data Fig. 4 [file 41586_2023_6836_MOESM1_ESM.pdf]

nature portfolio

https://doi.org/10.1038/s41586-023-06836-5

Supplementary information

Author Correction: Single-cell brain organoid screening identifies developmental defects in autism

In the format provided by the
authors and unedited

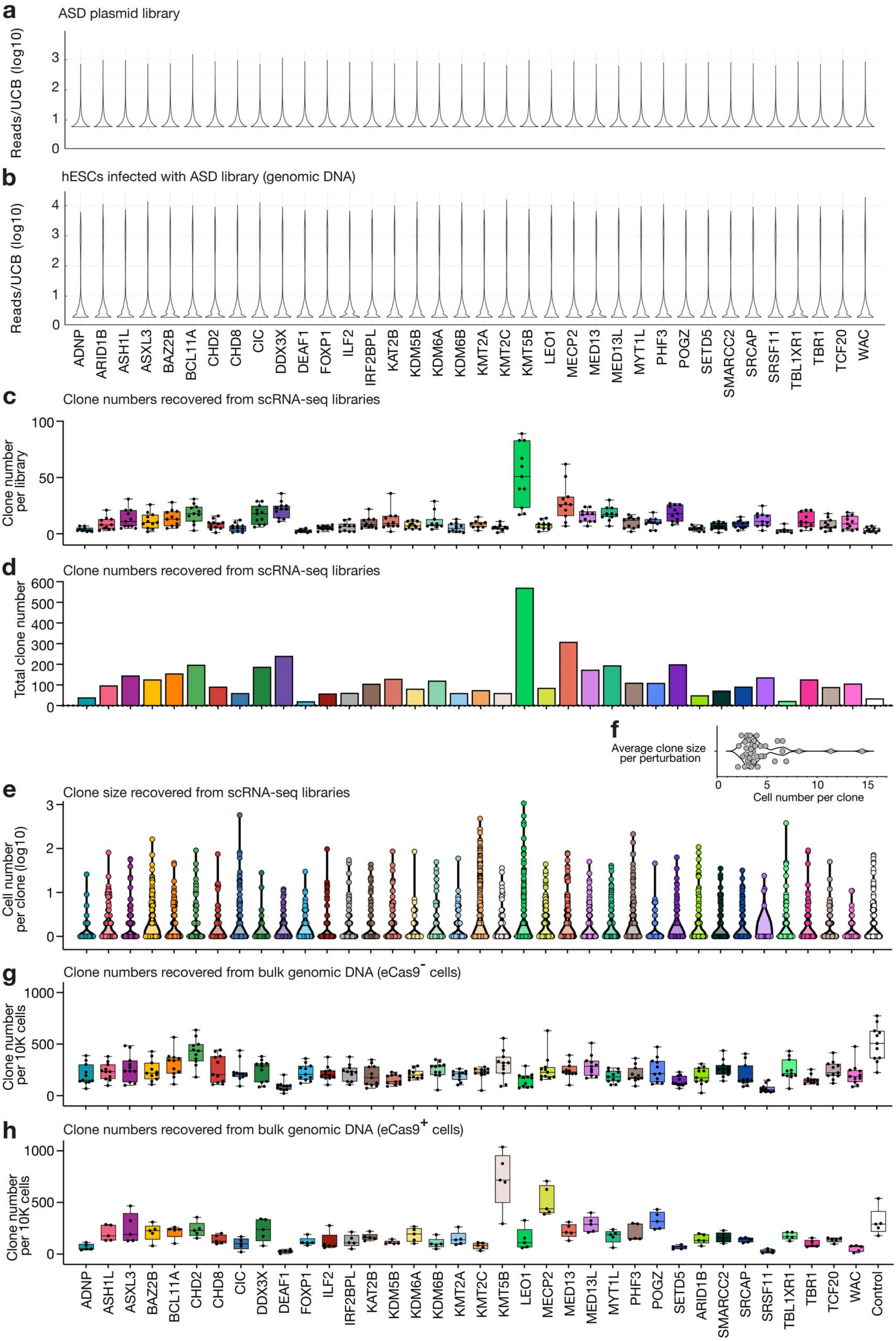

Original Extended Data Fig. 4

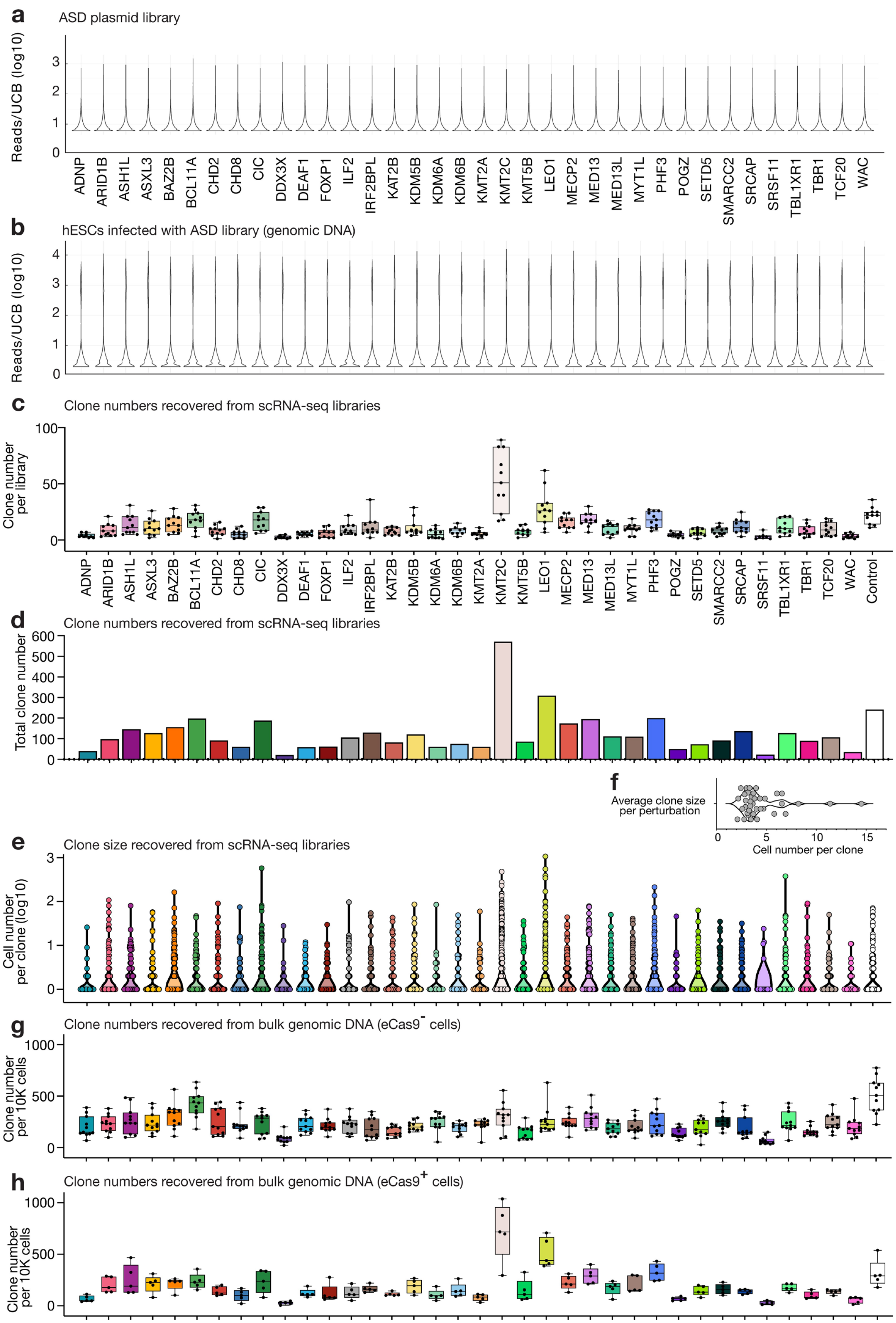

Revised Extended Data Fig. 4
